# Supplementary material for: Comparison of intramyocellular lipid metabolism in patients with diabetes and male athletes
Source: Nat Commun. 2024 May 15;15:3690. doi: 10.1038/s41467-024-47843-y (PMC11096352; doi:10.1038/s41467-024-47843-y)
Supplement: Supplementary file 1 — Supplementary Information [file 41467_2024_47843_MOESM1_ESM.pdf]

<u>Supplemental Table 1.</u> General characteristics of athletes and patients with type 2 diabetes mellitus enrolled in the study after screening.

	Athletes	***Patients with type 2 diabetes mellitus***
	**n=28**	**n=27**
Age (years)	52 ± 10	57 ± 8
Weight (kg)	75 ± 11	94 ± 14
Past Medical History		
Type 2 diabetes duration (years)	n/a	5.5
Hypertension	0	9
Hypercholesterolemia	0	17
Hypothyroidism	0	2
Previous renal colic	0	2
Diverticulosis	0	1
Mild asthma	0	2
Medication		
Metformin	0	19
Statins	0	17
Amlodipine	0	3
Angiotensin Receptor Blocker	0	5
International Physical Activity Questionnaire		
Total Walking (MET-minutes/week)	4080 ± 3383	2257 ± 2803

Total Moderate (MET-minutes/week)	4073 ± 2812	1675 ± 2093
Total Vigorous MET-minutes/week)	3981 ± 3197	1040 ± 2448
MET (minutes /week)	12371 ± 7444	4950 ± 6405
Sitting Total (minutes/week)	2003 ± 988	3284 ± 2174
Average Sitting Total (minutes/day)	286 ± 141	469 ± 310
7-Day Accelerometer activity levels		
Sedentary (minutes/day)	791 ± 151	905 ± 107
Light (minutes/day)	179 ± 58	157 ± 40
Moderate (minutes/day)	56 ± 15	29 ± 7
Vigorous (minutes/day)	26 ± 32	1 ± 1.4
Very Vigorous (minutes/day)	5 ± 12	0 ± 0.2
Average min in moderate/vigorous activity (minutes/day)	87 ± 40	30 ± 8
7-Day Food Diary		
Calories (Kcal)	11724 ± 4446	10326 ± 3609
Total fat (g)	465 ± 169	404 ± 182
Total protein (g)	478 ± 152	465 ± 137
Carbohydrates (g)	1387 ± 736	1233 ± 545
Sugar (g)	654 ± 506	389 ± 253
Starch (g)	719 ± 332	831 ± 397
Dietary fibre (g)	102 ± 39	95 ± 45

Alcohol (g)	59 ± 97	28 ± 46
Saturated fatty acids (g)	175 ± 88	139 ± 74
Monounsaturated fatty acids (g)	165 ± 57	152 ± 76
Poly-unsaturated fatty acids (g)	67 ± 24	70 ± 33
Cholesterol (mg)	1661 ± 1039	1450 ± 580
Echocardiography		
Left ventricular EDVi (mL/m2)	61 ± 11	40 ± 9
Left ventricular ESVi (mL/m2)	23 ± 6	14 ± 3
Left ventricular Ejection Fraction (%)	63 ± 6	65 ± 5
GLS (%)	-18.0 ± 1.5	-15.7 ± 2.5
Cardiopulmonary exercise testing		
RER	1.2 ± 0.1	1.2 ± 0.1
VO_2 peak (mL/min/kg)	45 ± 5	26 ± 3
VO_2 at AT (mL/min/kg)	32± 5	18 ± 2
VO_2/HR (ml/beat)	21 ± 3	16 ± 3
METs	13 ± 2	7± 1
Resting HR (bpm)	67 ± 10	77 ± 11
Peak HR (bpm)	160 ± 13	155 ± 13
Resting systolic BP (mmHg)	128 ± 9	133 ± 12
Resting diastolic BP (mmHg)	76 ± 9	80 ± 10
Peak systolic BP (mmHg)	172 ± 13	183 ± 15

Peak diastolic BP (mmHg)	85 ± 10	89 ± 6
VE/VCO_2 (slope)	27 ± 3	27 ± 3
Resting O_2 saturation (%)	99 ± 1	99 ± 1
Peak O_2 saturation (%)	97 ± 2	98 ± 1
FEV_1 (L)	4 ± 1	3 ± 1
Exercise duration (minutes)	9.5 ± 1.3	8.1 ± 1.1

Data are shown as mean ± SD. EDVi = end-diastolic volume index, ESVi = end-systolic volume index, GLS = global longitudinal strain, RER = respiratory exchange ratio, VO_2 = oxygen consumption, AT = anaerobic threshold, VO_2/HR = oxygen uptake per heartbeat or 'oxygen pulse', MET= metabolic equivalents, HR = heart rate, BP = blood pressure, VE/VCO_2 = ventilatory efficiency, FEV_1 = forced expiratory volume in first second. Unpaired t tests were used for all comparisons except the 7-day accelerometer data for which Mann-Whitney tests were used.

Supplemental Table 2. Nutritional analysis of the food diary of athletes and patients with type 2 diabetes mellitus at screening stage and repeated during the exercise intervention

	**Baseline Differences between Athletes and Type 2 diabetes patients**	**Athletes Change between Baseline and Deconditioning**	**Patients with Type 2 Diabetes Change between Baseline and Exercise Training**	**Difference in Changes between Athletes and Type 2 Diabetes patients**
Calories (Kcal)	1397 (-932 to 3728)	-301 (-2258 to 1657)	-908 (-2232 to 516)	607 (-1719 to 2933)
Total fat (g)	62 (-39 to 163)	12 (-85 to 110)	-53 (-125 to 19)	66 (-50 to 181)
Total protein (g)	13 (-71 to 97)	-22 (-97 to 54)	-35 (-99 to 29)	13.3 (-81.4 to 108)
Carbohydrates (g)	155 (-222 to 531)	-182 (-533 to 169)	-54 (-250 to 143)	-128 (-508 to 251)
Sugar (g)	265 (30 – 500)	-230 (-449 to -11)	0.2 (-120 to 121)	-230 (-466 to 5.14)
Starch (g)	-113 (-322 to 97)	59 (-105 to 223)	-49 (-140 to 42)	108 (-69 to 285)
Dietary fibre (g)	6.7 (-18 to 31)	-3.8 (-23 to 15)	-13 (-33 to 6.8)	9.4 (-17 to 36)
Alcohol (g)	31 (-14 to 76)	-14 (-62 to 35)	-12 (-38 to 14)	-2.1 (-54 to 50)
SFA (g)	36 (-11 to 84)	5 (-38 to 48)	-14 (-41 to 14)	19 (-30 to 68)
MONO (g)	13 (-25 to 52)	10.5 (-28 to 49)	-24 (-57 to 9)	34 (-14 to 83)
PUFA (g)	-3 (-20 to 14)	4 (-14 to 22)	-11 (-27 to 5)	15 (-8.3 to 38)
Cholesterol (mg)	211 (-282 to 703)	-173 (-728 to 381)	62 (-348 to 472)	-235 (-891 to 420)

Supplemental Figure 1: ^{1}H-Magnetic resonance spectroscopy of saturated and unsaturated extramyocellular lipid bonds. Data is shown as individual data points with means and error bars for standard deviation. Baseline and post-interventions comparisons were performed using t-tests.

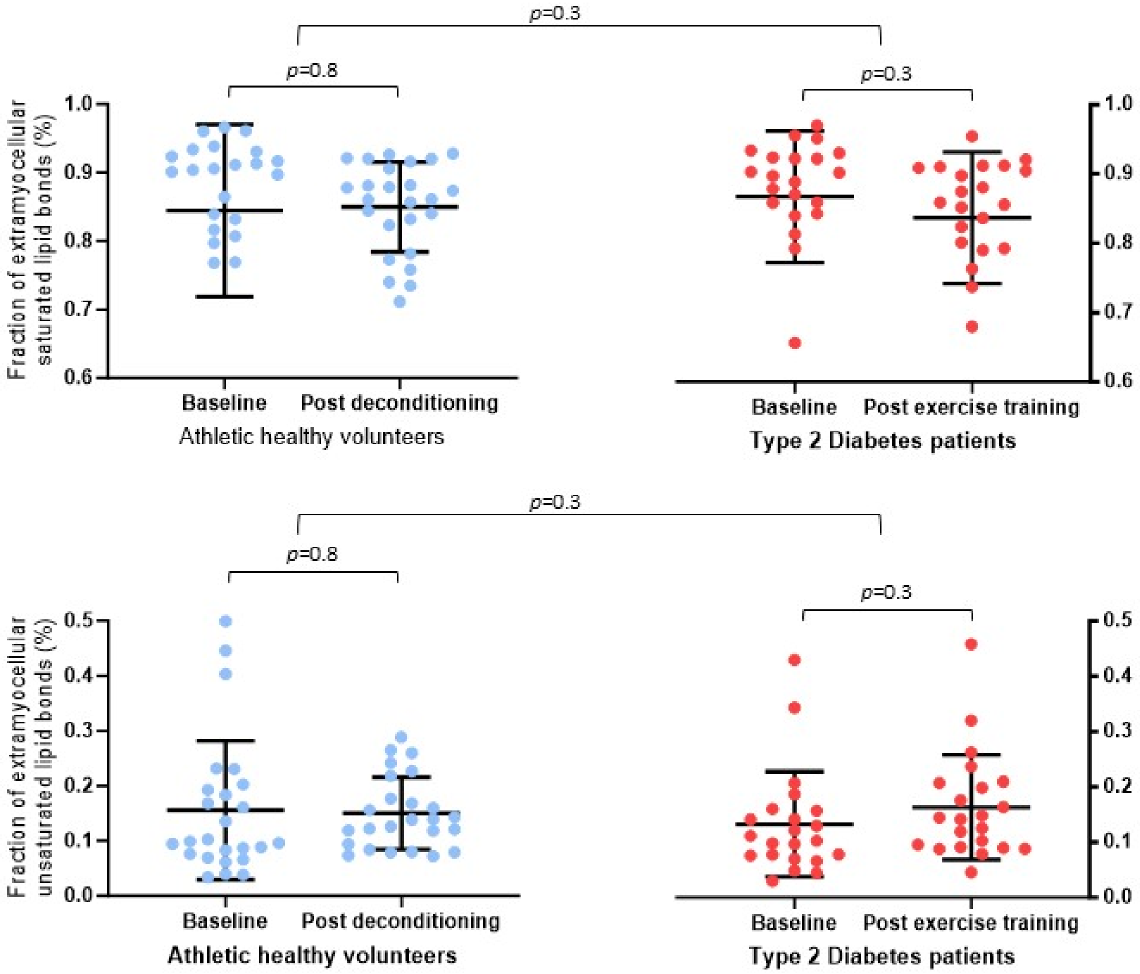

Supplemental Figure 2: ^{1}H-Magnetic resonance spectroscopy of saturated and unsaturated lipid bonds in adipose tissue. Data is shown as individual data points with means and error bars for standard deviation. Baseline and post-interventions comparisons were performed using t-tests.

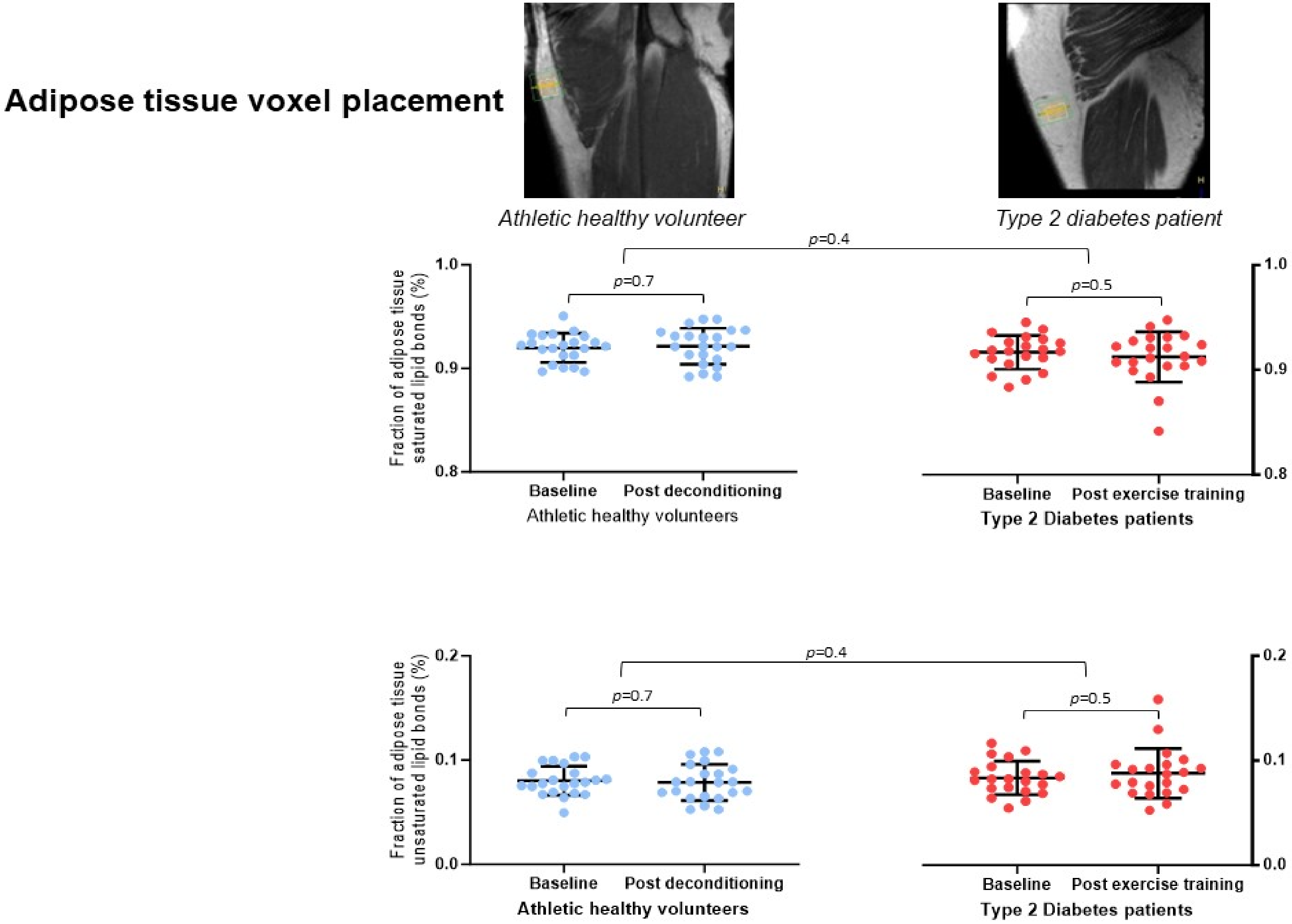

Comparison of intramyocellular lipid metabolism in patients with diabetes and male athletes

Alice M Mezincescu*[1], Amelia Rudd*[1], Lesley Cheyne[1], Graham Horgan[2], Sam Philip[1], Donnie Cameron[3], Luc van Loon[4], Phil Whitfield[5], Rachael Gribbin[6], May Khei Hu[1], Mirela Delibegovic[1], Barbara Fielding[6], Gerald Lobley[1], Frank Thies[1], David E. Newby[7], Stuart Gray[5], Anke Henning[8], Dana Dawson[1]

[1] Aberdeen Cardiovascular and Diabetes Centre, University of Aberdeen, Aberdeen, United Kingdom

[2] Biomathematics & Statistics Scotland, Aberdeen, United Kingdom

[3] C.J. Gorter MRI Center, Leiden University Medical Center, The Netherlands

[4] University of Maastricht, The Netherlands

[5] University of Glasgow, Glasgow, United Kingdom

[6] University of Surrey, United Kingdom

[7] Centre for Cardiovascular Science, University of Edinburgh, Edinburgh, United Kingdom

[8] Southwestern University, Texas, USA

Address for Correspondence:

Dana Dawson, Aberdeen Cardiovascular and Diabetes Centre, Polwarth Building, Foresterhill, University of Aberdeen, UK

Tel: +44 1224 559573, Fax: +44 1224 437971

Email: dana.dawson@abdn.ac.uk

∗ These authors contributed equally. The authors have declared no conflict of interest exists.

LIPIDOMICS MINIMAL REPORTING CHECKLIST

Separation Workflow

Created by https://lipidomicstandards.org, version v2.3.2

Overall study design

Title of the study	Comparison of intramyocellular lipid metabolism in patients with diabetes and male athletes

Document creation date	11/20/2023	Corresponding Email	Phil.Whitfield@glasgow.ac.uk
Principle investigator	Professor Dana Dawson	Is the workflow targeted or untargeted?	Untargeted
Institution	University of the Highlands and Islands	Clinical	Yes

Lipid extraction

Extraction method	2-phase system	2-phase system	Folch
pH adjustment	None	Were internal standards added prior extraction?	Yes

Analytical platform

Which solvents were used	Solvent A was H2O + 10 mM ammonium formate + 0.1 % (v/v) formic acid. Solvent B was IPA/ACN (9:1, v/v) + 10 mM ammonium formate + 0.1 % (v/v) formic acid	Ion source	ESI
Number of separation dimensions	One dimension	MS Level	MS1
Separation type 1	LC	Mass resolution for detected ion at MS1	High resolution
Separation mode 1 (liquid)	RP	Resolution at m / z 200 at MS1	100000
Detector	Mass spectrometer	Mass accuracy in ppm at MS1	5

MS type	Orbitrap	Was/were additional dimension techniques used	No
MS vendor	Thermo		

Quality control

Blanks	Yes	Quality control	Yes
Type of Blanks	Extraction blank, Injection blank	Type of QC sample	Sample pool

Method qualification and validation

Method validation	No

Reporting

Are reported raw data uploaded into repository?	No	Raw data upload	No
Are metadata available?	No	Additional comments	-

Sample Descriptions

Skeletal Muscle Biopsies / Human / Tissues (e.g., liver, heart, brain)

Perfusion	No	Storage time (month)	12
Provided information	Time to freeze (min), Storage time (month)	Additives	None
Temperature handling original sample	N2	Were samples stored under inert gas?	No
Instant sample preparation	No	Additional preservation methods	No
Time to freeze (min)	0	Biobank samples	No

Snap freezing in liquid N2	Yes	Sample homogenization	No
Storage temperature	-80 °C		

Lipid Class Descriptions

1) Cer[M+H]+ / Lipid identification

Lipid class	Cer	Check isomer overlap	No
MS Level for identification	MS1	RT verified by standard	Yes
Identification level	Species level	Separation of isobaric/isomeric interferece confirmed	No
Polarity mode	Positive	Model for separation prediction	No
Type of positive (precursor)ion	[M+H]+	Additional dimension/techniques	-
Isotope correction at MS1	No	Lipid Identification Software	Progenesis QI
MS1 verified by standard	Yes	Data manipulation	-
Background check at MS1	Yes	Nomenclature for intact lipid molecule	No
Did you presume assumptions for identification?	No	Further identification remarks	-

1) Cer[M+H]+ / Lipid quantification

Quantitative	Yes	Limit of quantification	No
MS Level for quantification	MS1	Normalization to reference	No
Internal lipid standard(s)	MS1	Lipid Quantification Software	Progenesis QI
Internal standard	Endogenous subclass		
Cer 18:1/17:0	Ceramides		
Type of quantification	Internal standard amount	Batch correction	No
Response correction	No	Further quantification remarks	-

Type 1 isotope correction	No

2) DG[M+Na]+ / Lipid identification

Lipid class	DG	Check isomer overlap	No
MS Level for identification	MS1	RT verified by standard	Yes
Identification level	Species level	Separation of isobaric/isomeric interferece confirmed	No
Polarity mode	Positive	Model for separation prediction	No
Type of positive (precursor)ion	[M+Na]+	Additional dimension/techniques	-
Isotope correction at MS1	No	Lipid Identification Software	Progenesis QI
MS1 verified by standard	Yes	Data manipulation	-
Background check at MS1	Yes	Nomenclature for intact lipid molecule	No
Did you presume assumptions for identification?	No	Further identification remarks	-

2) DG[M+Na]+ / Lipid quantification

Quantitative	Yes	Limit of quantification	No
MS Level for quantification	MS1	Normalization to reference	No
Internal lipid standard(s) MS1		Lipid Quantification Software	Progenesis QI

Internal standard	Endogenous subclass
DG 12:0/12:0	Diradylglycerols

Type of quantification	Internal standard amount	Batch correction	No
Response correction	No	Further quantification remarks	-
Type I isotope correction	No		

3) TG[M+NH4]+ / Lipid identification

Lipid class	TG	Check isomer overlap	No
MS Level for identification	MS1	RT verified by standard	Yes

Identification level	Species level	Separation of isobaric/isomeric interferece confirmed	No
Polarity mode	Positive	Model for separation prediction	No
Type of positive (precursor) ion	[M+NH4]+	Additional dimension/techniques	-
Isotope correction at MS1	No	Lipid Identification Software	Progenesis QI
MS1 verified by standard	Yes	Data manipulation	-
Background check at MS1	Yes	Nomenclature for intact lipid molecule	No
Did you presume assumptions for identification?	No	Further identification remarks	-

3) TG[M+NH4]+ / Lipid quantification

Quantitative	Yes	Limit of quantification	No
MS Level for quantification	MS1	Normalization to reference	No
Internal lipid standard(s) MS1		Lipid Quantification Software	Progenesis QI

Internal standard	Endogenous subclass
TG 17:0/17:0/17:0	Triradylglycerols

Type of quantification	Internal standard amount	Batch correction	No
Response correction	No	Further quantification remarks	-
Type I isotope correction	No		
