# Supplementary material for: Comparison of intramyocellular lipid metabolism in patients with diabetes and male athletes
Source: Nat Commun. 2024 May 15;15:3690. doi: 10.1038/s41467-024-47843-y (PMC11096352; doi:10.1038/s41467-024-47843-y)
Supplement: Supplementary file 3 — Reporting Summary [file 41467_2024_47843_MOESM3_ESM.pdf]

nature portfolio

Corresponding author(s): Professor Dana Dawson

Last updated by author(s): Mar 29, 2024

Reporting Summary

Nature Portfolio wishes to improve the reproducibility of the work that we publish. This form provides structure for consistency and transparency in reporting. For further information on Nature Portfolio policies, see our Editorial Policies and the Editorial Policy Checklist.

Statistics

For all statistical analyses, confirm that the following items are present in the figure legend, table legend, main text, or Methods section.

n/a	Confirmed	
☐	☒	The exact sample size (*n*) for each experimental group/condition, given as a discrete number and unit of measurement
☐	☒	A statement on whether measurements were taken from distinct samples or whether the same sample was measured repeatedly
☐	☒	The statistical test(s) used AND whether they are one- or two-sided<br>*Only common tests should be described solely by name; describe more complex techniques in the Methods section.*
☒	☐	A description of all covariates tested
☐	☒	A description of any assumptions or corrections, such as tests of normality and adjustment for multiple comparisons
☐	☒	A full description of the statistical parameters including central tendency (e.g. means) or other basic estimates (e.g. regression coefficient) AND variation (e.g. standard deviation) or associated estimates of uncertainty (e.g. confidence intervals)
☒	☐	For null hypothesis testing, the test statistic (e.g. *F*, *t*, *r*) with confidence intervals, effect sizes, degrees of freedom and *P* value noted<br>*Give P values as exact values whenever suitable.*
☒	☐	For Bayesian analysis, information on the choice of priors and Markov chain Monte Carlo settings
☒	☐	For hierarchical and complex designs, identification of the appropriate level for tests and full reporting of outcomes
☒	☐	Estimates of effect sizes (e.g. Cohen's *d*, Pearson's *r*), indicating how they were calculated

Our web collection on statistics for biologists contains articles on many of the points above.

Software and code

Policy information about availability of computer code

Data collection	The data was collected as described in detail in the methods section. The demographic, food diary, and self reported activity levels data was collected via self administered questionnaires on paper then transferred into a Microsoft Excel spreadsheet.
Data analysis	All echocardiography images obtained were then analysed offline using the commercially available software EchoPAC version 2.01 (GE Healthcare, United Kingdom).<br>All food diary data was analysed using the commercially available software WinDiets version 1.0 (Robert Gordon University, UK).<br>Self-reported physical activity data was analysed using the IPAQ scoring protocol available from its developer: https://sites/google.com/site/theipaq/<br>Accelerometer data was analysed using the commercially available software ActiLife version 6.13.3 (ActiGraph LLC, USA).<br>Cardio-pulmonary exercise testing data was collected and analysed using the commercially available software Quark PFT (Cosmed, Italy).<br>All Cardiac magnetic resonance image analysis was performed using commercially available software CMRTools (Cardiovascular Solutions, London, UK).<br>All 1H magnetic resonance spectroscopy data were analysed using the 'Muscle-5' setting in the commercially available (free) software LC Model Version 6.3 (Stephen Provencher, Oakville, ON, Canada).<br>Western-blot relative quantification of protein bands was obtained using the public domain available software image J version 1.52p (NIH and LOCI, USA).<br>All statistical data analysis was done using IBM SPSS Statistics 29.0.1.0

For manuscripts utilizing custom algorithms or software that are central to the research but not yet described in published literature, software must be made available to editors and reviewers. We strongly encourage code deposition in a community repository (e.g. GitHub). See the Nature Portfolio guidelines for submitting code & software for further information.

Data

Policy information about availability of data

All manuscripts must include a data availability statement. This statement should provide the following information, where applicable:

- Accession codes, unique identifiers, or web links for publicly available datasets
- A description of any restrictions on data availability
- For clinical datasets or third party data, please ensure that the statement adheres to our policy

The datasets generated and/or analysed during the current study that can be shared after they are fully anonymised to comply with GDPR, and are available from the corresponding author on reasonable request, within one year of online publication.

Research involving human participants, their data, or biological material

Policy information about studies with human participants or human data. See also policy information about sex, gender (identity/presentation), and sexual orientation and race, ethnicity and racism.

Reporting on sex and gender	All participants were of male sex by study design, as intramyocellular lipid amount and kinetics is known to be significantly different between sexes, therefore for a mechanistic investigation of limited size we could only obtain funding for subjects of one sex only.
Reporting on race, ethnicity, or other socially relevant groupings	We included all comers according to inclusion criteria. The north of Scotland is not an ethnically diverse region of the country. Thus, whilst the majority of the participants were Caucasian, we had representation from the Asian and black African ethnic communities.
Population characteristics	Male sex, of the same age distribution, matched within +/-5 years as recruitment of participants occurred simultaneous in both groups.
Recruitment	Endurance trained athletic healthy volunteers were recruited by advertising the study in a variety of public places (health clubs) and by personal contact within local and regional running, triathlon and cycling clubs, placing approved posters at local gyms and fitness clubs or by asking various sports organisations and clubs to advertise the research invite poster on their social media channels. The potential volunteers who came forward and met the eligibility criteria were invited for screening. Most of the type 2 diabetes patients were recruited from the local general practice database, identifying those who had a diagnosis of type 2 diabetes who met the inclusion/exclusion criteria for the study. Those identified as eligible received an invitation letter in the post. Patients who were willing and appeared eligible to participate were then invited for screening. A smaller number of volunteers were recruited through displaying a type 2 diabetes patient recruitment poster in a variety of public places in Aberdeen and through advertisement in the local press and via a local radio station advert.
Ethics oversight	The study presented herewith received ethical approval from the North of Scotland Research Ethics Committee (REC reference number 16/NS/0024) on 22nd March 2016. Registration with NHS Grampian Research & Development Office and approval to proceed were received on 11th May 2016. The study was registered on clinicaltrials.gov on the 12th September 2016 by the researchers but due to the unfilled research governance officer post at the University of Aberdeen, this was not dealt with until the 7th December 2016, followed by a delay incurred at the clinicaltrials.gov website who finally launched it live on the 27th February 2017. All participants gave written informed consent to participate in the study.

Note that full information on the approval of the study protocol must also be provided in the manuscript.

Field-specific reporting

Please select the one below that is the best fit for your research. If you are not sure, read the appropriate sections before making your selection.

☒ Life sciences ☐ Behavioural & social sciences ☐ Ecological, evolutionary & environmental sciences

For a reference copy of the document with all sections, see nature.com/documents/nr-reporting-summary-flat.pdf

Life sciences study design

All studies must disclose on these points even when the disclosure is negative.

Sample size	The sample size was powered to detect changes in the 1H MRS as the primary end-point. For this, each subject served as their own control for changes observed after their respective exercise intervention. Sample size calculations were done based on prior work of one of the applicants (AH) which reported skeletal muscle MRS peaks of saturated/unsaturated fatty acid bonds of 500±170mM/l and 60±20mM/l (mean ± SD) respectively in healthy subjects. We aimed to detect a 10% change in saturated and unsaturated intramyocellular peaks after exercise training intervention. Thus, the minimum number of participants was calculated at n=25 subjects in each group for a study power of 0.8 with an alpha of 0.05.
Data exclusions	n=3 athletic healthy volunteers and n=5 type 2 diabetes patients 1H magnetic resonance spectra was excluded from the analysis. Poor quality of spectroscopy data recorded with motion artefact and thus no clear separation of intramyocellular and extramyocellular lipid peaks could

	not be analysed as distinct lipid resonances could not be resolved and quantified in the commercially available software (LC Model).
Replication	Replication of data acquisition was not done as it is not appropriate for such intense human studies and it was not part of the protocol or ethical approval. However, each participant was their own control before and after the intervention.
Randomization	This study was non-randomised by design.
Blinding	This study was not blinded as this was not possible by design.

Reporting for specific materials, systems and methods

We require information from authors about some types of materials, experimental systems and methods used in many studies. Here, indicate whether each material, system or method listed is relevant to your study. If you are not sure if a list item applies to your research, read the appropriate section before selecting a response.

Materials & experimental systems

n/a	Involved in the study
☐	☒ Antibodies
☒	☐ Eukaryotic cell lines
☒	☐ Palaeontology and archaeology
☒	☐ Animals and other organisms
☐	☒ Clinical data
☒	☐ Dual use research of concern
☒	☐ Plants

Methods

n/a	Involved in the study
☒	☐ ChIP-seq
☒	☐ Flow cytometry
☒	☐ MRI-based neuroimaging

Antibodies

Antibodies used	To avoid duplication, these are all listed in Supplemental Table 2.
Validation	All antibodies were tested in several replicates on human skeletal muscle that was surplus to diagnostic requirements and we had ethical approval to use for optimisation experiments.

Clinical data

Policy information about clinical studies

All manuscripts should comply with the ICMJEguidelines for publication of clinical research and a completedCONSORT checklist must be included with all submissions.

Clinical trial registration	https://clinicaltrials.gov/ct2/show/NCT03065140
Study protocol	The study protocol is presented at the link above, otherwise it can be sourced from the University of Aberdeen Research Governance Office upon request.
Data collection	The study data was collected between February 2017 and end of June 2019 at the University of Aberdeen.
Outcomes	The primary outcome measure was non-invasive measurement of saturated and unsaturated lipids in the vastus lateralis muscle by 1H magnetic resonance spectroscopy.<br>Secondary outcome measure was the saturated and unsaturated lipid pool turnonver (kinetics) assessed by 4 hour-intravenous infusions of stable isotopes combined with pre and post infusion skeletal muscle biopsies for determination of muscle enrichments.

Plants

Seed stocks	N/A
Novel plant genotypes	N/A
Authentication	N/A
