# Supplementary material for: Comparison of intramyocellular lipid metabolism in patients with diabetes and male athletes
Source: Nat Commun. 2024 May 15;15:3690. doi: 10.1038/s41467-024-47843-y (PMC11096352; doi:10.1038/s41467-024-47843-y)
Supplement: Supplementary file 4 — Soure Data [file 41467_2024_47843_MOESM4_ESM.zip › MISTY Full unedited gel for Figure 3 -R4-NCOMMS-23-12748.docx]

**MISTY Western Blot Ponceau**


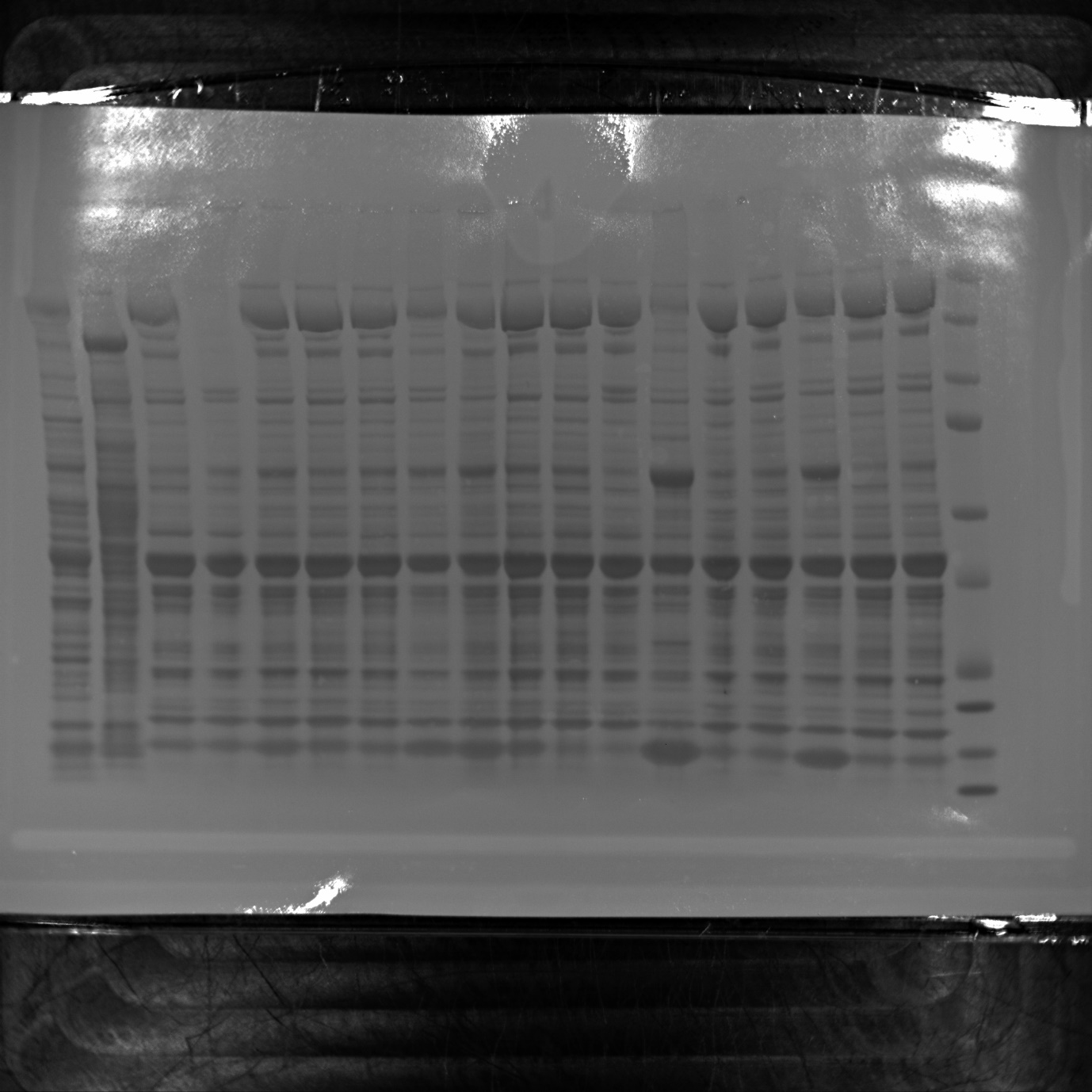


t/p AMPK/AKT

Ires 1

pIR

CPT1B

Glut1/4

pERK

PS6

GAPDH

Expected band size

Ires 1 180kDa

CPT1B 88kDa

p/t-AKT 60kDa

pERK 42kDa

pIR 100kDa

p/tAMPK 62-64kDa

PS6 32kDa

GAPDH 37kDa


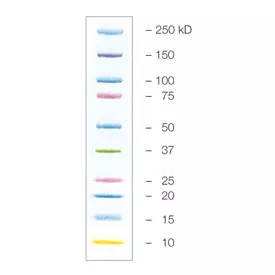


Cutting the membranes

Gel 1

Cut just above 100kDa, for Ires1

Cut through 75kDa, between this cut and the last for CPT1B

Cut through 50kDa this upper section for pAKT

Bottom section for pERK and also GAPDH (striped and re-probed after)

Gel 2

Cut through 75kDa, upper part for pIR 1162

Cut at 50kDa upper part for pAMPK

Cut at 20kDa upper part for PS6 and GAPDH (re-probe)

Lower portion for FABP3

Gel 3

Cut at 20kDa and 75kDa this portion for Glut 1/Glut 4 and GAPDH (re-probe).

Gel 4

As Gel 3 but other antibody

| Lane | No |  | Sample |
| --- | --- | --- | --- |
|  |  |  |  |
| L1 | athlete 1 |  | V1 |
|  |  |  |  |
| L2 | athlete 1 |  | V1 |
|  |  |  |  |
| L3 | athlete 1 |  | V2 |
|  |  |  |  |
| L4 | athlete 1 |  | V2 |
|  |  |  |  |
| L5 | DM 1 |  | V1 |
|  |  |  |  |
| L6 | DM 1 |  | V1 |
|  |  |  |  |
| L7 | DM 1 |  | V2 |
|  |  |  |  |
| L8 | DM 1 |  | V2 |
|  |  |  |  |
| L9 | athlete 2 |  | V1 |
|  |  |  |  |
| L10 | athlete 2 |  | V1 |
|  |  |  |  |
| L11 | athlete 2 |  | V2 |
|  |  |  |  |
| L12 | athlete 2 |  | V2 |
|  |  |  |  |
| L13 | DM 2 |  | V1 |
|  |  |  |  |
| L14 | DM 2 |  | V1 |
|  |  |  |  |
| L15 | DM 2 |  | V2 |
|  |  |  |  |
| L16 | DM 2 |  | V2 |
|  |  |  |  |
| L17 | rat liver |  | +ve Control |
|  |  |  |  |
| L18 | rat heart |  | +ve Control |

MISTY Western Blots pAKT, tAKT, GAPDH

GEL 1 - pAKT

ladder


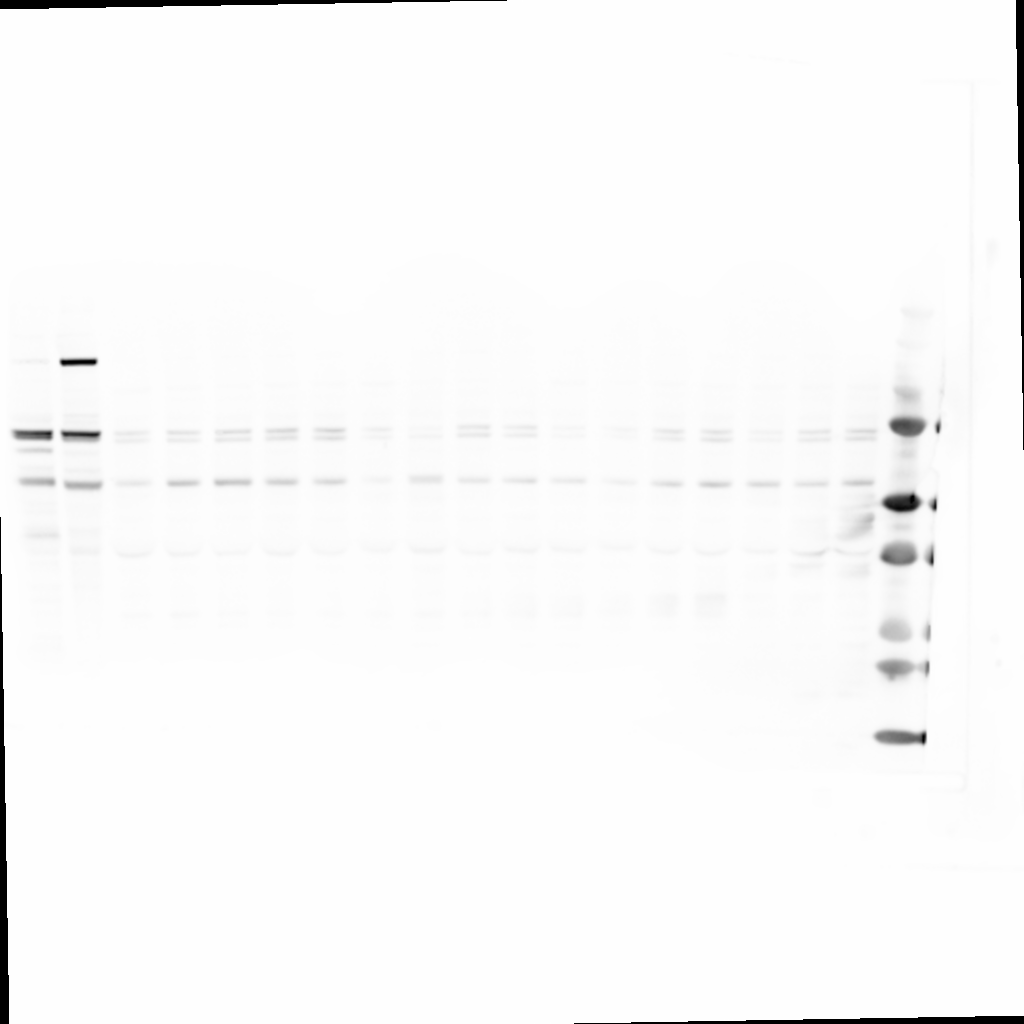


L11

L12

L13

L14

L15

L16

L17

L18

L10

L9

L8

L7

L6

L5

L4

L3

L2

L1

pAKT

GEL 1 – tAKT

ladder


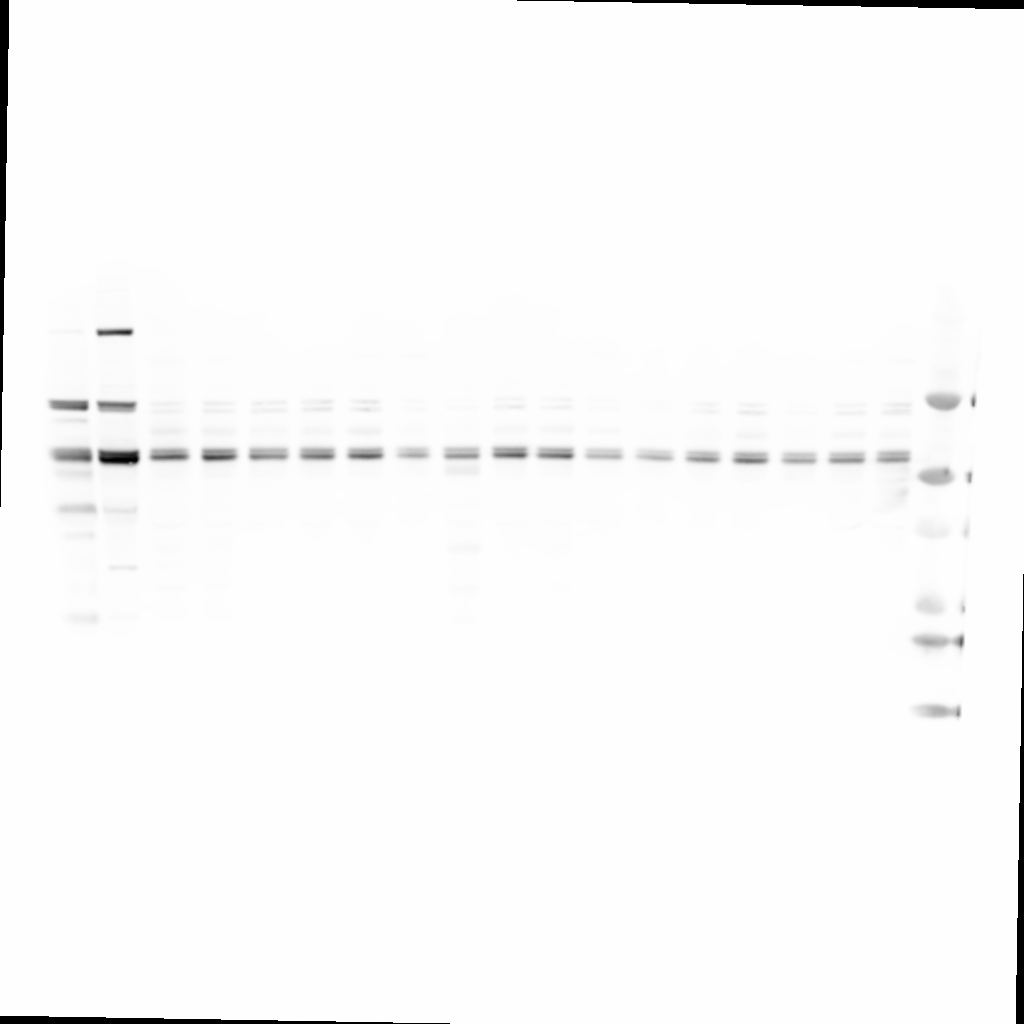


L1

L2

L10

L9

L8

L7

L6

L5

L4

L3

L18

L17

L16

L15

L14

L13

L12

L11

tAKT

GEL 1 – GAPDH


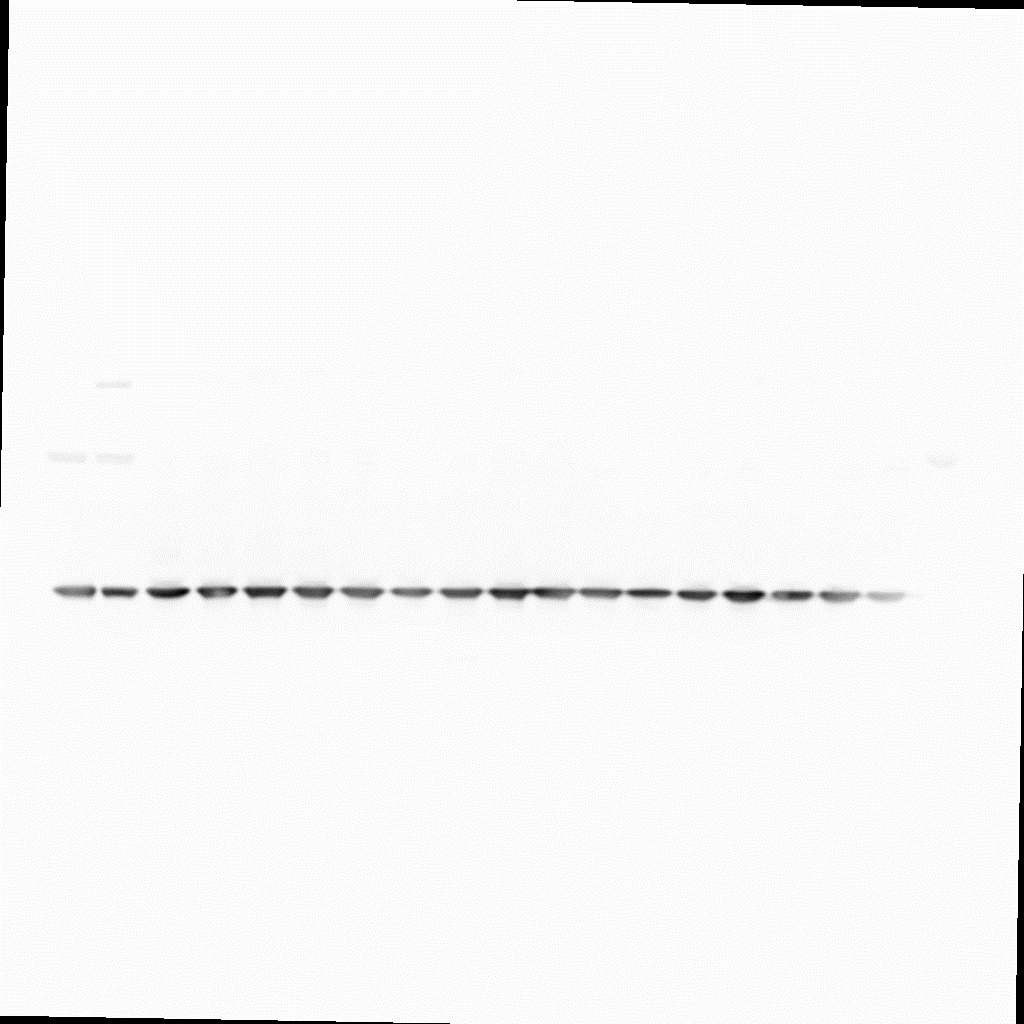


ladder

ladder

L1

L2

L10

L9

L8

L7

L6

L5

L4

L3

L18

L17

L16

L15

L14

L13

L12

L11

GAPDH

GEL 2 – pAMPK


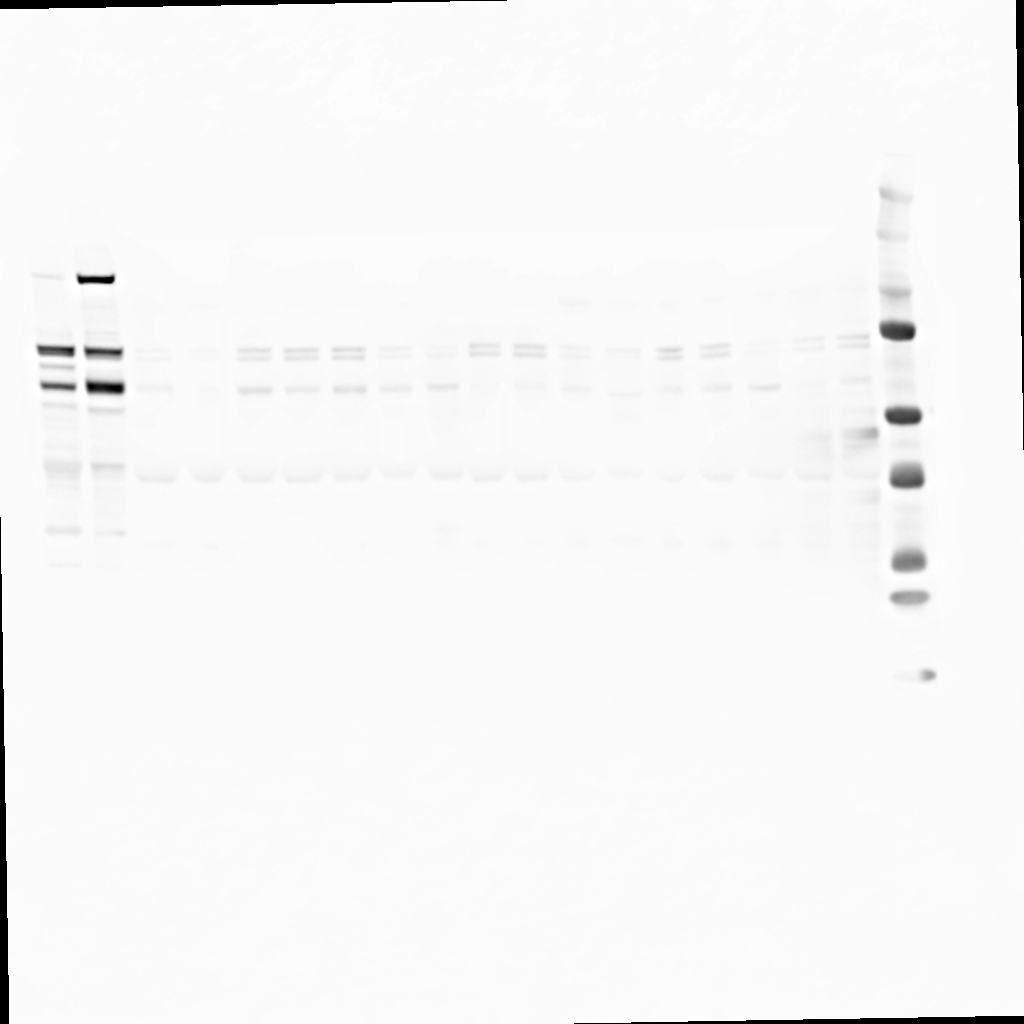


L1

L2

L9

L8

L7

L6

L5

L4

L3

L18

L17

L16

L15

L14

L13

L12

L11

L10

pAMPK

GEL 2 – tAMPK


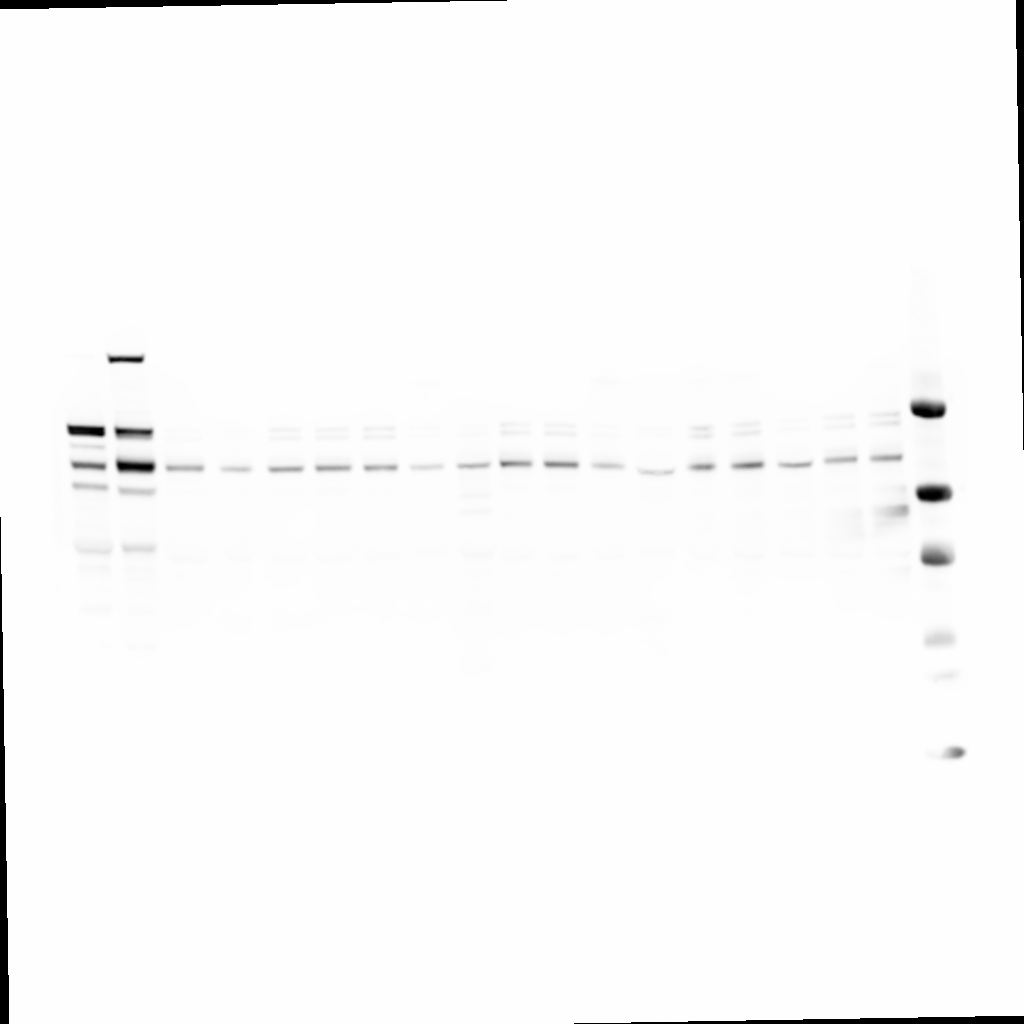


ladder

L1

L2

L9

L8

L7

L6

L5

L4

L3

L18

L17

L16

L15

L14

L13

L12

L11

L10

tAMPK

GEL 2 – GAP-DH

ladder


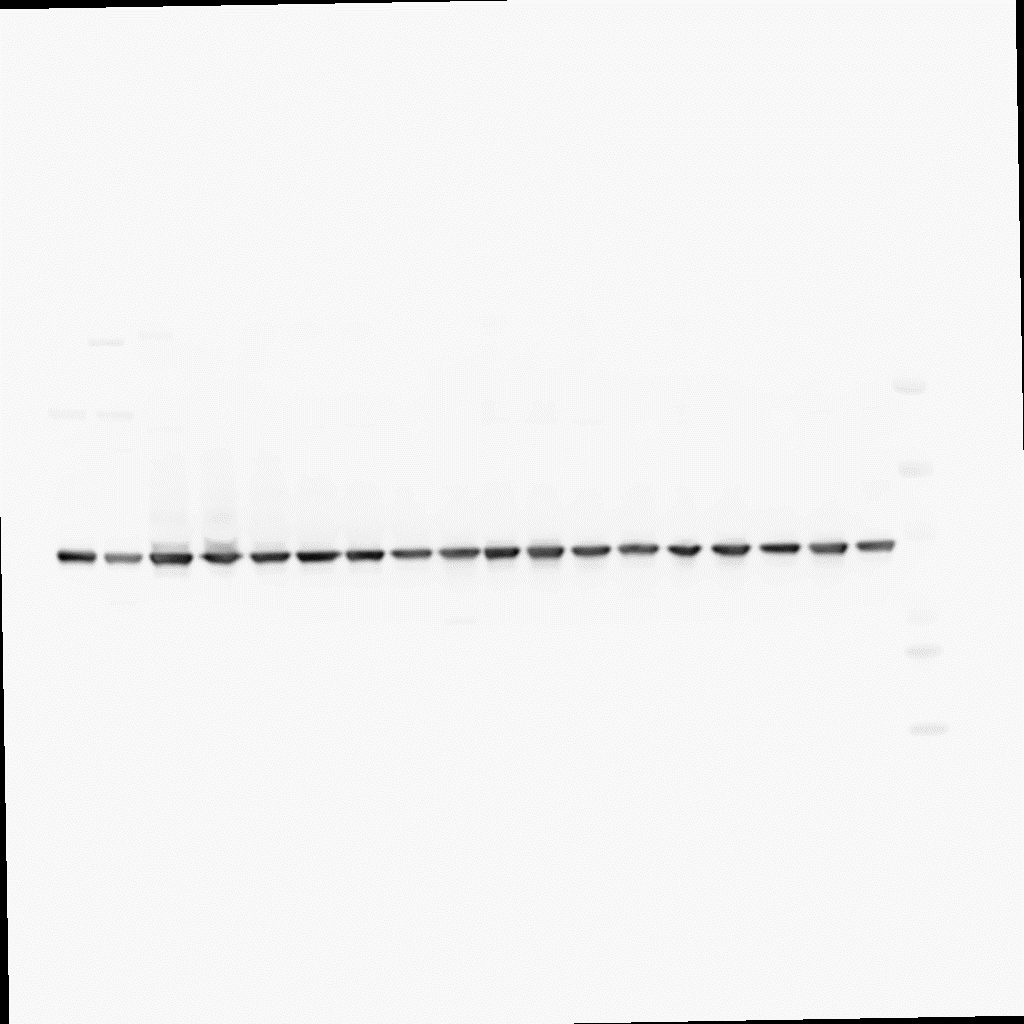


L1

L2

L9

L8

L7

L6

L5

L4

L3

L18

L17

L16

L15

L14

L13

L12

L11

L10

GAPDH
